# Cold Knife Versus Carbon Dioxide for the Treatment of Preinvasive Cervical Lesion

**DOI:** 10.3390/medicina60071056

**Published:** 2024-06-27

**Authors:** Federico Ferrari, Emma Bonetti, Giulia Oliveri, Andrea Giannini, Elisa Gozzini, Jacopo Conforti, Filippo Alberto Ferrari, Federica Salinaro, Giancarlo Tisi, Giuseppe Ciravolo, Alessandro Favilli, Franco Odicino

**Affiliations:** 1 Department of Clinical and Experimental Sciences, University of Brescia, 25136 Brescia, Italy; federico.ferrari@unibs.it (F.F.); e.bonetti007@unibs.it (E.B.);; 2 Unit of Gynecology, “Sant’Andrea” Hospital, Department of Surgical and Medical Sciences and Translational Medicine, Sapienza University of Rome, 00189 Rome, Italy; 3 Department of Obstetrics and Gynaecology, AOUI Verona, University of Verona, 37126 Verona, Italy; 4 S.C. Ostetricia e Ginecologia, ASST Spedali Civili Brescia, Dipartimento Area Della Donna e Materno Infantile, 25136 Brescia, Italy; 5 Department of Medicine and Surgery, University of Perugia, 06123 Perugia, Italy

**Keywords:** cervical cancer, cold knife conization, carbon dioxide laser conization

## Abstract

*Background and Objectives:* Cervical cancer (CC) represents a significant health concern worldwide, particularly for younger women. Cold knife (CK) conization and carbon dioxide (CO_2_) laser conization are two techniques commonly used to remove pre-invasive lesions, offering a potential curative intent in cases of incidental diagnosis of CC. This study aimed to assess the clinical implications and pathological outcomes of CK vs. CO_2_ laser conization for pre-invasive lesions. *Materials and Methods:* We retrospectively analyzed women who underwent CO_2_ or CK conization for high-grade preinvasive lesions (CIN2/3, CIS and AIS) between 2010 and 2022. Patient demographics, surgical details and pathological outcomes were collected. Pregnancy outcomes, including composite adverse obstetric rates, and oncological follow-up data, were also obtained. *Results:* In all, 1270 women were included; of them, 1225 (96.5%) underwent CO_2_, and 45 (3.5%) underwent CK conization. Overall, the rate of positive endocervical or deep margins was lower with CO_2_ laser compared to CK (4.3% vs. 13.3%, *p* = 0.015). Incidental CC was diagnosed in 56 (4.4%) patients, with 35 (62.5%) squamous and 21 (46.6%) adenocarcinomas. In a multivariate regression model, the relative risk for positive endocervical or deep margins is significantly greater in cases of incidental diagnosis of CC (*p* < 0.01). In cases of incidental diagnosis of CC, we found that the probabilities of having either positive endocervical or deep margins after CO_2_ laser or CK conization are similar, with a higher risk in case of adenocarcinoma lesion. Among women with CC, 42 (75%) opted for radical treatment, while 14 (25%) underwent a follow-up. Only one woman (7.1%) in the follow-up group, who had undergone CK conization, experienced a composite adverse obstetric outcome. No recurrences were observed after a median follow-up of 53 months. *Conclusions:* CO_2_ laser conization achieved a lower positive margin rate overall. CK and CO_2_ conization appear to be equivalent oncological options for incidental CC.

## 1. Introduction

Cervical cancer (CC) is the fourth-most-frequent cancer in women worldwide, with around 660,000 new cases in 2022 [1]. Furthermore, in the United States, it ranks as the second cause of cancer-related death among women aged 20 to 39 years [2]. Squamous cell carcinoma is the most prevalent histological type globally, accounting for approximately 80% of CC cases. Adenocarcinoma follows closely behind, representing roughly 12% of all CC diagnoses [3].

Since human papillomavirus (HPV) has been identified as a CC causative agent and the progression from precancerous lesions to invasive cancer is usually slow, CC is one of the most preventable cancers [4]. The implementation of widespread screening programs in recent years has demonstrably improved early-detection rates, leading to a rise in the number of CC survivors. This success is reflected in the declining prevalence of the locally advanced disease and a slight increase in the diagnosis of early-stage cancers. Moreover, it is noteworthy that an estimated 40% of early-stage CC patients are younger than 40 years old [5].

Radical hysterectomy is considered the standard treatment for early-stage CC, but it results in permanent infertility. In young women with childbearing desire, fertility preservation acquires a paramount importance [5,6].

Cervical conization is a standard surgical procedure that allows the removal of cervical lesions, and it has been widely accepted for treating FIGO stage IA1 CC [7,8,9]. A study by the U.S. Surveillance, Epidemiology and End Results (SEER) program specifically analyzed women under 40 with stage IA1 CC. The study found no significant difference in the 5-year survival rates between those who received conization alone and those who underwent demolitive treatment [9].

While colposcopic-directed cervical biopsy remains a crucial diagnostic tool for cervical intraepithelial neoplasia (CIN), a phenomenon known as pathological upgrading can occur in clinical practice. This situation refers to cases in which the definitive diagnosis from a conization specimen reveals a more severe lesion compared to the initial biopsy. This discrepancy is more prevalent in postmenopausal women, due to lower estrogen levels, squamocolumnar junction recession and cervical atrophy. The overall incidence of pathological upgrading is estimated to be around 10% in women diagnosed with CIN 2 or 3 [10].

Several techniques exist for performing cervical conization, with cold knife (CK) conization, carbon dioxide (CO_2_) laser conization, and loop electrosurgical excision procedure (LEEP) being the most common. However, there is a scarcity of high-quality comparative studies on these methods.

Although the LEEP remains the first-line treatment recommended by the major international societies [7,11], CO_2_ laser conization seems to be a viable alternative for treating CIN, demonstrating favorable outcomes regarding specimen resection margins and the obstetric prognosis [12]. Retrospective studies comparing conservative treatments for adenocarcinoma in situ (AIS) using CK conization or LEEP have shown a potential advantage for CK conization, with lower margin positivity rates [13,14].

Given the limited recent data comparing CK and CO_2_ laser conization, our present study aims to assess the comparative effects of these two techniques on clinical implications and pathological outcomes.

## 2. Materials and Methods

This is a single-center retrospective study conducted at our tertiary care center associated with the University of Brescia, Italy. We retrospectively enrolled patients who underwent CK or CO_2_ laser conization at our Division of Obstetrics and Gynecology at ASST Spedali Civili of Brescia between January 2010 and December 2022.

All data used in this article derive from an auditing of the databases used in the service of evaluations for cervical cancer and is hence already anonymized at the moment of data analysis. The design, analyses and interpretations of data, and the drafting and revisions conform to the Helsinki Declaration, the Committee on Publication Ethics guidelines (http://publicationethics.org/, accessed on 1 March 2023) and the Reporting of Studies Conducted Using Observational Routinely-Collected Health Data (RECORD) Statement, as validated by the Enhancing the Quality and Transparency of Health Research Network (http://www.equator-network.org, accessed on 1 March 2023). No personal data that could lead to actual identification of a patient were stored in the databases. The study was not advertised.

The inclusion criteria consisted of the following: (i) age ≥ 18 years and treatment naive; (ii) newly diagnosed CIN2 or higher and AIS or higher, obtained by colposcopic biopsy; and (iii) surgical excision performed with either CO_2_ laser or CK conization, the decision of the technique being left to the discretion of the surgeon. Exclusion criteria were: (i) CIN1 or negative colposcopic biopsy; and (ii) any case of invasive cancer.

Patient demographics and medical history were collected, including the age at the time of conization, parity (number of term pregnancies), menopausal status, and preoperative histological biopsy (CIN2 of higher, CIS and AIS). Surgical data were also retrieved, including date of the surgical procedure, type of conization (CO_2_ laser or CK conization), and type of anesthesia (local or general).

The definitive histological results from the conization specimen were collected, including the following: postoperative histological report (negative, CIN 1, CIN2, CIN3, CIS, squamous cell cancer, AIS or adenocarcinoma); presence of stromal invasion; exocervical margin status (negative or positive); endocervical/deep margin status (negative or positive); and, eventually, FIGO stage (according to FIGO 2009 staging system) for cases diagnosed with cervical cancer. Negative or positive margins were defined according to our institutional technique, which has been already published [15]. In fact, further ablative treatment with CO_2_ or monopolar was adopted for exocervical and endocervical margins for at least 5–8 mm to ensure the radicality of the treatment, determining a cylindrical footprint in the cervix. We reported treatment or follow-up modality in cases of incidental diagnosis of CC. Since most CC diagnoses in our study predated the implementation of the 2018 FIGO staging system revision, we restaged all the cases according to the most recent staging system.

Although different surgeons performed the procedures, the quality of care remained consistent throughout the study period, as previously described in a published work [15].

For women diagnosed with invasive CC who chose follow-up instead of immediate treatment, we assessed clinical endpoints based on established safety criteria, including recurrence rates and obstetrics outcomes [16,17].

We considered a follow-up period of up to 5 years. Follow-up included a Pap smear, a colposcopy if indicated by the Pap smear results, and a comprehensive gynecological examination. We classified patients as lost to follow-up if we lacked documented follow-up data after their surgical procedure.

To assess obstetric outcomes, we investigated the following data points: childbearing desire (yes or no); pregnancy rate (number of pregnancies achieved after the procedure); delivery mode (vaginal delivery or cesarean section); and obstetric complications, including need for prophylactic cerclage (yes or no), preterm delivery (yes or no), and preterm premature rupture of membranes (yes or no). Spontaneous miscarriages in the first trimester were excluded from the analysis due to the lack of association with conization procedures [18].

Given the low prevalence of obstetric complications in our population, we opted to analyze them as composite adverse obstetric outcomes. This measure comprehensively included the presence of any of the aforementioned obstetric complications.

Our primary objective was to compare the surgical margin positivity rates between CO_2_ laser and CK conization for all types of cervical lesions.

The secondary objectives were to investigate, in women with incidental diagnosis of CC, the impacts of the two conization techniques on surgical margin status, recurrence rates and clinical implication, and to evaluate the composite rate of adverse obstetric outcomes in women who opted for follow-up after treatment.

### Statistical Methods

The selected population was described using median and interquartile range (IQR) for data which exhibited a non-normal distribution. Absolute and relative frequencies were used for other categorical variables. The Mann–Whitney U test was performed to compare the median between subgroups, as the data showed a non-normal distribution. The existence of any association between categorical variables was investigated using chi-squared tests or Fisher’s exact test, if necessary.

A multivariate analysis was conducted through a binomial logistic regression, considering as the dependent variable the presence of a positive deep margin (dichotomous) and as independent variables the presence of a preoperative CC diagnosis (dichotomous), the presence of a postoperative CC squamous histology (dichotomous), the presence of a postoperative CC adenocarcinoma histology (dichotomous), and conization type (CK vs. CO_2_ laser conization); these were all categorical variables. Logistic regression allows the ascertainment of the simultaneous effects of the independent variables on the likelihood that patients show positive deep margins. Predictive effects for all independent variables are obtained from logistic regression coefficients in terms of odds ratios, and statistical significance for all tests was set at *p* = 0.05, while statistical analysis was conducted using IBM SPSS Statistics (version 26) software.

## 3. Results

A total of 1270 patients met the inclusion criteria and were enrolled in this study. The study population consisted of 1225 patients (96.5%) who underwent CO_2_ laser conization and 45 patients (3.5%) who underwent CK conization.

### 3.1. Patient Characteristics

Table 1 summarizes the characteristics of the patients included in the study, classified according to the conization technique.

The median age at surgery was similar between two groups, 38 years (IQR 31–46) for CO_2_ laser and 38.6 years (IQR 33.5–48) for CK conization (*p* = 0.321). Local anesthesia was most commonly adopted for CO_2_ laser (92.4%), while general anesthesia was more frequent for CK conization (97.8%).

### 3.2. Primary Objective

Our primary objective was to assess the rate of positive endocervical or deep margins between the CO_2_ laser and CK conization groups for all types of cervical lesions, and the analysis revealed a statistically significant difference (*p* = 0.015), with a lower rate of positive margins in the CO_2_ laser group (4.3%) compared to CK group (13.3%), as can be seen in Table 1. Overall, the rates of positive exocervical margins were not statistically significant (*p* > 0.05). Interestingly, a strong association (*p* < 0.001) between the type of preoperative histology and the positivity of the endocervical or deep margins (Table 2) was noted.

The distribution of preoperative diagnoses also differed significantly between the two conization groups (*p* < 0.001). In the CO_2_ laser group, the most frequent preoperative diagnosis was cervical intraepithelial neoplasia of grade 3 or lower (≤CIN3), at 93.1%. Conversely, the CK group showed a higher prevalence of adenocarcinoma in situ (AIS).

### 3.3. Secondary Objectives

According to planned secondary aims, of 1270 cases, we identified 56 cases (4.4%) diagnosed with invasive CC after the conization procedure. Of them, 35 (62.5%) were squamous cell carcinomas and 21 (37.5%) adenocarcinomas. Overall, 34 underwent CO_2_ laser conization and 22 underwent CK conization, as detailed in Table 3 and Table 4.

The diagnosis of adenocarcinoma was significantly less frequent in the CO_2_ laser conization group when compared to CK conization (17.6% vs. 54.5%; *p* < 0.01).

The maximum size of CC showed a non-normal distribution for cases treated with both types of conizations. The median for CO_2_ laser was 1.5 mm (IQR 0.5–11) and for CK 2.5 mm (IQR 0.5–11), with no significant difference observed using the Mann–Whitney U test (*p* = 0.60).

FIGO 2018 staging is shown in Table 5. The distribution of stages between the two procedures did not differ significantly (*p* = 0.19).

The rate of positive endocervical or deep margins is shown in Table 6. In this analysis, the conization technique significantly affected the margin positivity, with a higher rate observed in the CO_2_ laser group when compared to the CK group (58.8% vs. 22.7%, with *p* < 0.01).

Instead, with multivariate analysis (Table 7), we found that regardless of the surgical technique, postoperative incidental diagnoses of CC for both squamous and adenocarcinoma histology were independent predictors of positive endocervical or deep positive margins, while the type of lesion reported in the preoperative setting was not statistically significant.

Patients treated with CK conization had an odds ratio of 0.28 for positive endocervical/deep margins compared to those treated with CO_2_ laser conization, although statistical significance was not achieved (*p* = 0.06).

After a median follow-up of 53 months (IQR 32–76), no recurrences of cervical cancer were recorded in patients treated with either CO_2_ laser or CK conization, regardless of FIGO 2018 stage.

Of the 56 patients diagnosed with invasive carcinoma, 42 (75%) underwent radical treatment and 14 (25%) opted for exclusive follow-up, due to their desire for pregnancies. This latter subgroup included seven patients who underwent CO_2_ laser conization and seven who underwent CK conization. The prevalence of composite adverse obstetric outcomes was very low, with only one case (7.1%) observed, in a patient who underwent CK conization.

## 4. Discussion

In our retrospective analysis, the cervical conization using CO_2_ laser offers, overall, a lower rate of positive endocervical or deep margins when compared to CK conization. In cases of incidental diagnosis of CC, we found that the probability of having either positive endocervical or deep margins after CO_2_ laser or CK conization is the same. There is a higher risk of having positive endocervical or deep margins in incidental adenocarcinoma when compared to squamous carcinoma, regardless of the conization technique.

Incomplete excision of cervical lesions, defined by positive endocervical or deep surgical margins, is a concern, since it can lead to residual disease and impact future treatment decisions [19,20]. Therefore, our primary objective was to evaluate margin status. While no significant difference emerged in exocervical margin positivity between the techniques, the CO_2_ laser conization showed a lower rate of positive endocervical or deep margins, compared to CK conization, for all types of cervical lesions (4.3% vs. 13.3%). This finding aligns with a 2017 meta-analysis by Arbyn et al. [21] which considered the margin status for three methods (LEEP, CO_2_ laser and CK conization), demonstrating a lower positive margin rate for CO_2_ laser (17.8%) compared to CK (20.2%) across both endo- and exocervical margins. They also observed no significant change in positive margin rates for either technique over a considerable time frame (1975–2016).

These results are further supported by Bogani et al. (2020) [22] who investigated LEEP and CO_2_ laser in relation to positive margins and reported a higher prevalence with LEEP (11.2%) compared to CO_2_ laser (4.2%), particularly for endocervical margins (6% vs. 2.2%). However, a recent study by Mosseri et al. (2024) [23] found no association between surgical method (LEEP vs. laser excision) and margin positivity.

Interestingly, our study revealed a significant association between preoperative diagnosis and margin positivity. In fact, 91.7% of patients with negative margins had a preoperative histological diagnosis of CIN3 or lower. This suggests that the risk of positive margins might be lower for cervical intraepithelial neoplasia compared to more advanced lesions (namely, CIS and AIS). Of course, conversely, the difference in preoperative diagnoses may explain the observed higher rate of positive margins in the CK group with a higher rate of AIS lesions.

However, the relationship between disease severity and margin involvement is inconsistent in the literature. While Shin et al. reported no such association [24], Costa et al. found disease severity to be the most significant factor [25]. Similarly, Arbyn et al. observed a rising rate of incomplete excision with increasing lesion severity [21]. These contrasting findings suggest the need for further investigation into the factors influencing margin positivity.

Interestingly, among the identified incidental cases of CC, no recurrences were observed, regardless of the conization technique. However, the type of conization significantly impacted margin involvement (*p* < 0.01), with a higher rate of positive margins observed in the CO_2_ laser group compared to the CK group, contrasting with our results across the whole population. The diagnosis of adenocarcinoma was significantly more frequent (*p* < 0.01) in the CK conization group (68.2%) compared to CO_2_ laser conization (17.6%), but this discrepancy can reflect the preoperative histological selection bias. The postoperative incidental diagnosis of CC (either squamous or adenocarcinoma) significantly increased the odds of having positive endocervical or deep margin rates, regardless of the surgical technique. This result can be interpreted in the context of a missed diagnosis of CC in the preoperative setting, potentially with a lesion inside the endocervical canal. Interestingly, patients treated with CK conization had a lower odds ratio for positive endocervical/deep margins, compared to those treated with CO_2_ laser conization. Colposcopy plays a crucial role in diagnosing cervical diseases. Biopsy guided by colposcopy provides a histological diagnosis, which is essential for assessing the most appropriate treatment. However, a key limitation of colposcopy is the biopsy area, which depends on whether the entire lesion is visible and accessible. As the transformation zone migrates deeper into the cervical canal with age and menopause, complete removal of the entire lesion becomes more challenging. Consequently, a negative preoperative biopsy may miss a deeper CC component within the canal.

In our clinical practice, CO_2_ laser conization is the most frequently employed technique due to its several advantages. First, it can be performed in an outpatient setting, without the need for general anesthesia. Additionally, the CO_2_ laser offers advantages in terms of pain reduction and superior hemostasis due to its tissue-coagulating properties.

According to our findings, it seems that surgeons prefer reserving the use of CK conization for specific cases, particularly those with a preoperative diagnosis of AIS, as can be seen in Table 1. This preference can arise because of the well-known deeper location of AIS lesions within the cervical canal and because, with the use of CK conization, the surgeon can remove completely the endocervical part of the cervix. Based on our findings regarding the lower odds of overall positive endocervical or deep margins with CK conization, further studies specifically investigating the broader application and long-term outcomes of CK conization are warranted, and we cannot now conclude that CK conization might be preferable when colposcopy cannot explore the cervical canal even if the cranial margin of the lesion is not visible.

Importantly, we must consider the impact of conization on reproductive potential. Studies suggest that CK conization may be associated with higher rates of obstetric complications, such as preterm delivery, premature rupture of membranes and lower birth weight [26,27]. However, due to the limited sample size in our study, including only one case of CK conization with a composite adverse obstetric outcome, further research with a larger cohort of women is necessary to definitively assess the association between CK conization and these obstetric complications.

The limitations of our study include its retrospective and single-center design, introducing potential selection bias and limiting the generalizability of our findings; in fact, this is not a randomized clinical trial. Another substantial limitation is the unbalanced proportion of women undergoing CO_2_ laser vs. CK conization, and hence, generalization to the overall population can suffer by this selection bias. Additionally, the analysis did not evaluate lesion depth, multifocality or HPV testing status, and long-term obstetrical outcomes were unavailable, as well as records of pap smear status at the moment of colposcopy.

Future studies employing prospective, multicenter, randomized controlled trials are needed to address these limitations and assess more robustly the impacts of conization technique on clinical and obstetrical outcomes. These future studies should also take into account the triaging pap smear and HPV testing status obtained before colposcopy and use these elements of data to build a decisional algorithm or score to select the most appropriate excisional treatment.

## 5. Conclusions

To our knowledge, this is the first study comparing CO_2_ laser and CK conization in preinvasive lesions of the cervix. A lower rate of positive endocervical or deep margins when using CO_2_ laser conization is observed, as compared to CK conization. In cases of incidental diagnosis of CC, we found that the probability of having either positive endocervical or deep margins after CO_2_ laser or CK conization is the same. A higher risk of having positive endocervical or deep margins in incidental adenocarcinoma was seen when compared to squamous carcinoma, regardless of the conization technique.

## Figures and Tables

**Table 1:** Characteristics of the 1270 patients who underwent CO_2_ laser and CK conization.

	Laser CO_2_ (n = 1225)	CK (n = 45)	*p*
Age (median, IQR)	38 (31–46)	38.6 (33.5–48)	0.321
Parity			0.459
Nullipara	592 (48.3%)	12 (26.7%)	
Multiparae	633 (51.7%)	17 (37.8%)	
Missing	-	16 (35.5%)	
Postmenopause			<0.001
No	1185 (96.7%)	36 (80.0%)	
Yes	40 (3.3%)	9 (20.0%)	
Preoperative histology			<0.001
≤CIN3	1140 (93.1%)	9 (20.0%)	
CIS	71 (5.8%)	6 (13.3%)	
AIS	14 (1.1%)	30 (66.6%)	
Postoperative histology			<0.001
≤CIN3	1072 (87.5%)	11 (24.4%)	
CIS	101 (8.2%)	2 (4.4%)	
Squamous cell carcinoma	28 (2.3%)	6 (13.3%)	
AIS	18 (1.5%)	14 (31.1%)	
Adenocarcinoma	6 (0.5%)	12 (26.7%)	
Anesthesia			<0.001
Local	1132 (92.4%)	1 (2.2%)	
General	93 (7.6%)	44 (97.8%)	
Exocervical margin			0.07
Negative	1035 (84.5%)	29 (64.4%)	
Positive Missing	190 (15.5%) -	12 (26.6%) 4 (8.8%)	
Endocervical or deep margin			0.015
Negative	1172 (95.7%)	39 (86.7%)	
Positive	53 (4.3%)	6 (13.3%)	

**Table 2:** Comparison of preoperative histology and endocervical or deep margin status for all types of cervical lesions at preoperative histology.

		Endocervical or Deep Margins	
		Negative	Positive
Preoperative histology			
	≤CIN3	1111 (91.7%)	38 (64.4%)
	CIS	64 (5.3%)	13 (22%)
	AIS	36 (3%)	8 (13.5%)
Total		1211 (100%)	59 (100%)

**Table 3:** Comparison of preoperative and postoperative histology in patients with incidental cervical cancer treated with CO_2_ laser conization.

		Postoperative Histology		
		Squamous Carcinoma	Adenocarcinoma	
Preoperative histology				
	≤CIN3	21 (84%)	4 (16%)	25 (73.5%)
	CIS	7 (87.5%)	1 (12.5%)	8 (23.5%)
	AIS	0	1 (100%)	1 (2.9%)
		28 (82.4%)	6 (17.6%)	34 (100%)

**Table 4:** Comparison of preoperative and postoperative histology in patients with incidental cervical cancer treated with CK conization.

		Postoperative Histology		
		Squamous Carcinoma	Adenocarcinoma	
Preoperative histology				
	≤CIN3	3 (100%)	0	3 (13.6%)
	CIS	4 (100%)	0	4 (18.2%)
	AIS	0	15 (100%)	15 (68.2%)
		6 (27.3%)	12 (54.5%)	22 (100%)

**Table 5:** FIGO staging distribution.

		CO_2_ Laser	CK
FIGO 2018 Stage			
	IA1	18 (52.9%)	12 (54.6%)
	IA2	0 (0.0%)	1 (4.5%)
	IB1	13 (38.2%)	7 (31.8%)
	IB2	2 (5.8%)	1 (4.5%)
	IIA2	0 (0.0%)	1 (4.5%)
	IIIC1	1 (2.9%)	0 (0.0%)
		34 (100%)	22 (100%)

**Table 6:** Endocervical or deep margin status distribution in women with incidental CC.

		CO_2_ Laser	CK
Endocervical or deep margin			
	Negative	14 (41.2%)	17 (77.3%)
	Positive	20 (58.8%)	5 (22.7%)
Total		34 (100%)	22 (100%)

**Table 7:** Logistic regression analysis of endocervical or deep margin assessment.

	OR	CI 95%	*p*
Preoperative diagnosis of squamous lesion	1.76	0.33–9.45	0.51
Preoperative diagnosis of AIS	1.89	0.45–78.2	0.63
Postoperative incidental diagnosis of squamous cancer	39.0	17.25–88.22	<0.01
Postoperative incidental diagnosis of adenocarcinoma	59.7	15.3–232.36	<0.01
CK conization (vs. CO_2_)	0.28	0.072–1.09	0.06

## Data Availability

The data presented in this study are available on request from the corresponding author. The data are not publicly available due to privacy or ethical restrictions.
